# PEG-J replacement for duodenal levodopa infusion in Parkinson’s disease patients: a retrospective study

**DOI:** 10.1186/s12883-021-02546-5

**Published:** 2022-01-13

**Authors:** Simone Simoni, Pasquale Nigro, Marta Filidei, Giulia Cappelletti, Federico Paolini Paoletti, Danilo Castellani, Mirko Gaggiotti, Lucilla Parnetti, Nicola Tambasco

**Affiliations:** 1 Movement Disorders Center Neurology Department, Perugia General Hospital University of Perugia, P.le Severi 1, 06132 Perugia, Italy; 2 Neurology Department, Perugia General Hospital University of Perugia, Perugia, Italy; 3 Endoscopic Section of Gastroenterology and Hepatology, Perugia General Hospital and University of Perugia, Perugia, Italy

**Keywords:** Duodenal levodopa infusion, PEG-J, PEG-J replacement, Parkinson’s disease

## Abstract

**Background:**

Reducing percutaneous endoscopic gastrostomies with jejunal extension tubes (PEG-J) related complications is vital to the long-term preservation of duodenal levodopa infusion (DLI) in advanced Parkinson’s disease (APD). Here, we provide data on the frequency of complications for both the standard “pull” and the non-endoscopic, radiologic assisted, *“*push*”* replacement PEG-J techniques in APD.

**Methods:**

We retrospectively identified all patients treated with DLI from October 2009 to January 2020 at the Movement Disorders Center. Patients features and demographics, PEG-J procedures, causes for any discontinuation, reported complications and mortality were collected. In this cohort, PEG-J replacements were performed using the standard “pull” procedure or the radiologic assisted “push” method. Descriptive statistical analysis, t-test and paired t-test with False Discovery Rate correction was performed.

**Results:**

This retrospective study included 30 APD patients [median age 72 ± 5.6 years; mean disease duration 17.2 + 5.7 years]. Mean treatment duration was 35.6 (30.6) months. Overall, 156 PEG-J procedures were performed, and Nineteen patients (63.3%) had a total of 185 reported complications, 85 of which were peristomal complications. 17 (56.6%) underwent 100 replacement procedures due to complications. The most commonly reported complication for replacement was J-tube dislocation (36%). One patient discontinued treatment after 6 months, due to peripheral neuropathy. Six patients died for causes not related to DLI. PEG-J replacements performed with the “push” method had a higher turnover (5.6 vs. 7.6 mo.), but fewer reported complications (67 vs. 75%).

**Conclusion:**

The overall rate of complications was lower for “push” technique. This result might have been due to a higher replacement turnover that acted as a protective factor.

## Background

**Fig. 1:**
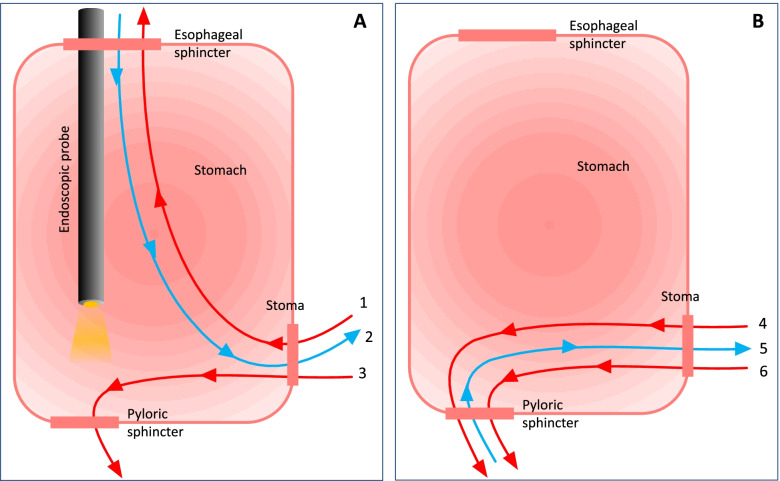
(**A**) Scheme illustrating the «pull» technique.1 Transesophageal removal of old PEG-J via endoscopy.2 New gastric tube, connected to a guide wire, is *pulled* from stoma under gastroscopy.3 Endoscopic placement of jejunal tube, (**B**) Scheme illustrating the «push» technique. 4 Insertion of a guide wire into the lumen of the old PEG-J.5 After deflation ofthe internal bumper, the old PEG-J is removed from the stoma, while the wire is kept in situ.6 New PEG-J is fed over the wire*,* through stoma, under fluoroscopic guidance.The figure is our own.

Duodenal levodopa infusion (DLI) is a widely utilized treatment for patients with advanced Parkinson’s disease (APD). [1] It provides continuous levodopa infusion directly into the proximal jejunum by way of percutaneous endoscopic gastrostomy with jejunal extension tube (PEG-J) connected to a portable infusion pump. [2] DLI overcomes slow and erratic gastric emptying, producing more consistent levodopa plasma levels, generally leading to a significant improvement in on-time without troublesome dyskinesia, a reduction in off-time, and a concomitant improvement of quality of life. [3] PEG-J was first described in 1998, and has become a reliable technique for setting the tubing for DLI. [2] Long-term safety studies [4] and recommendations for best practice in PEG-J placement [5] have reduced procedural and post-procedural adverse events rates. However, during the course of DLI, both scheduled, and emergency PEG-J replacements (due to PEG-J obstruction, looping, phytobezoars, or buried bumper syndrome) can occur. [6] Both settings may expose patients to repeated endoscopic procedures, which may negatively influence compliance and easily lead to discontinuation. [5, 7] PEG-J placements and, in most of cases, replacements are gastroscopic procedures carried out in an endoscopy room by two gastroenterologists, an anesthesiologist and two specialized nurses. To render the replacement procedure less invasive for the patient, sedation is required, [5] utilizing intravenous infusion of propofol or midazolam, due to their fast onset of action and short half-life. After the PEG-J is placed, an abdomen X-ray is performed to verify the correct position of the J-tube. An alternative technique, only suitable for tubes replacement, and not for the first implants, is also performed at the Endoscopic Department of Gastroenterology at Perugia University Hospital for patients with DLI. This so-called “push” technique requires a softer PEG-J, with an internal water-inflated balloon instead of a silicone bumper and is performed under fluoroscopy guidance avoiding the need for an endoscopy. The replacement tube is inserted using a guide wire that is led through the old tube before it is removed. No hospitalization, further radiological follow-up or sedation are required. Both procedures are outlined in Fig. 1. The aim of this study was to provide data on the frequency of complications for both the standard “pull” and the non-endoscopic, radiologic assisted, “push” replacement PEG-J techniques in APD patients receiving DLI.

## Methods

This observational retrospective study was carried out to collect data from APD patients treated with DLI at the our Movement Disorders Center. All reviewed files were of patients who met diagnostic criteria for PD [8] and who had undergone DLI between October 2009 and January 2020. All patients had undergone a standardized evaluation at first PEG-J placement (baseline). Data regarding age, sex, weight, PD duration, Unified Parkinson’s Disease Rating Scale (UPDRS) part III score, modified Hoehn and Yahr (mH&Y) stage, PEG-J model, and DLI doses were collected. For the purpose of this study, different data from follow-up visits was also analyzed: reason for replacement, complications, replacement procedure utilized, PEG-J model, UPDRS part III, *m*H&Y stage, DLI doses and causes of withdrawal. The last follow-up visit was recorded in January 2020.

Descriptive statistical analysis was performed for collected data. A t-test, with False Discovery Rate (FDR) correction, was performed to compare all clinical features for “pull” and “push” techniques. A paired t-test with with False Discovery Rate (FDR) correction was performed to compare scheduled and unexpected replacements was performed.

The study conformed to the Declaration of Helsinki guidelines and was approved by the local Ethics Committee Comitato Etico regionale (CER) Umbria (n. 4032/19).

## Results

Of the 30 patients demographic and clinical features are reported in Table 1. Overall patients underwent a total of 156 PEG-J procedures, of which 20 patients had undergone at least one PEG-J replacement procedure, with a mean follow-up period of 64,4 (3–119) months, and an average of 4 replacements. Only one patient discontinued DLI due to the occurrence of a severe peripheral neuropathy. Over the follow-up period, six patients died, and the reported causes of death were unrelated to DLI. The total mean follow-up was of 21,4 months, with a range of 3 to 54 months. During a 12 months observation period, 14 patients out of 30 (46,7%) reported at least one complication. 8 patients (26,7%) complained about a stoma condition; 11 patients (13,3%) underwent 22 PEG-J replacement procedures due to complications, of which 15 were associated with the tubing system utilized, and 7 with patient related issues. In a 10-year follow-up, nineteen patients (63.3%) had a total of 185 complications, 85 of which were related to stoma issues: stomal erythema (*n* = 44), serous secretions (*n* = 20), surgical wound granuloma (*n* = 9), peristomal edema (*n* = 6), purulent secretions (*n* = 3), sero-ematic secretions (*n* = 2), candida infection (*n* = 1). 17 patients (56.6%) underwent a total amount of 100 PEG-J replacements due to complications, of which 82 out of 100 were associated with the tubing system utilized [tube dislocation (*n* = 35), external or internal bumper dislocation (*n* = 21), tube breaking/puncture (*n* = 16), device coloring (*n* = 8), accidental removal (*n* = 1), candida colonization of the tubing system (*n* = 1)], while the remaining 18 with patient related issues [abdominal discomfort (*n* = 9),

**Table 1:** Demographics, clinical features, PEG-J procedures and related complications

***n*=30**							
Male	60%						
Age at disease onset (y)	53.9	(35-62)	± 6.8				
Age at PEG-J placement	69.4	(51-80)	± 6.4				
Age at LFV (y)	724	(61-82)	± 5.6				
Disease duration (y)	17.2	(10-33)	± 5.7				
Disease duration at PEG-J placement (y)	14.9	(6-28)	± 6.3				
Follow-up duration (mo)	61.3	(3-119)	± 32.6				
Active follow-up	23						
Patients acquired from other sites	3						
Deceased	6						
Discontinued	1						
Patients with 23 (11-38) ± 8.2 months of follow-up (*n*=20)							
	*baseline*				*LFV*		
MDS-UPDRS pt. III score	31.6	(4-50)	± 14		33.5	(8-66)	± 15.2
mH&Y stage	3	(2.5-4)	± 0.5		3	(2.5-4)	± 0.6
Weight (kg)	57.5	(38-76)	± 10.8		57	(36-75)	± 11.3
Morning dose (ml)	7.8	(3.6-12.5)	± 2.7		8.1	(3.6-12.6)	± 1.8
Continuous infusion (ml/h)	3.1	(2-4.3)	± 0.7		3.3	(2-4.6)	± 0.8
Extra dose (ml)	2.2	(1.3-3.5)	± 0.6		2.2	(0.5-3.5)	± 0.8
Average extra doses per day	1.2	(0-3)	± 1.0		1.2	(0-5)	± 1.1
Daily DLI dose (mg)	1072	(710-1486)	± 236.4		1152	(708-1698)	± 262
**PEG-J procedures (*n*=156)**							
First implants	30						
Scheduled replacements	53						
Not scheduled (unexpected) replacements	73						
**Complications related PEG-J replacements**	Events	Patients					
**Device-related**							
Accidental removal	1	1					
Device coloring	8	4					
Tube breaking/puncture	16	6					
Ext./int. bumper dislocation	21	9					
Candida colonization	1	1					
Tube dislocation	35	12					
**Patient-related**							
Abdominal discomfort	9	5					
Granulation tissue	4	4					
Buried bumper syndrome	1	1					
Peristomal inflammation	4	4					
**Total**	100						
**Stoma complications**^a^							
Candida infection	1	1					
Granuloma	9	3					
Erythema	44	13					
Serous secretions	20	3					
Sero-ematic secretions	2	1					
Purulent secretions	3	1					
Edema	6	2					
**Total**	85						

Abbreviations: *DLI* duodenal levodopa infusion, Ext./int. = external or internal, *LFV* last follow-up visit, *mH&Y* modified Hoehn and Yahr stage, *PEG-J* percutaneous endoscopic gastrostomy with jejunal extension tube, *UPDRS* Unified Parkinson’s Disease Rating Scale. Data are mean values and percentage of total (n) or (range) ± standard deviation (*SD*).

^a^More than one complication can be reported for each visit.

granulation tissue (*n* = 4), peristomal inflammation (*n* = 4), buried bumper syndrome (*n* = 1)] (Table 1). The most common reason given for replacement was J-tube dislocation (36%) in 12 patients (Table 1). A correlation analysis between complications and clinical scales (UPDRS and H&Y) during times of replacement did not find any significant values. However, the small number of patients and the retrospective design of the study could have influenced the results.

The “pull” method was adopted for 41 procedures (from 2009 to 2014) and the “push” method for 79 (from 2015 to 2019). Complications led to tube replacement in 75% of cases with “pull” and 67% of cases with “push” technique. Mean PEG-J duration was of 7,6 and 5,6 months for the “pull” and “push” methods, respectively. A comparison of clinical features for “pull” and “push” technique is reported in Table 2: a t-test (FDR corrected) showed no significant differences for age, disease duration, follow-up duration, UPDRS III score, mH&Y score and PEG-J duration. The paired t-test to compare scheduled replacements and unexpected replacements groups was significant (*p* < 0.05), however, after the FDR correction for variability did not showed this result due to the low number of subjects for each group (Table 2).

**Table 2:** PEG-J replacement procedures: “pull” and “push” methods comparison

	**“pull” *n*=41**			**“push” *n*=79**		
**Clinical characteristics**^b^	Mean	range	SD	Mean	range	SD
Age (years)	72.6	63-84	± 7.0	73,2	63-84	± 6.6
Disease duration (years)	19.8	15-35	± 5.7	18.6	12-35	± 5.7
Follow-up duration (months)	77.4	3-119	± 4.4	64.9	15-119	± 3.4
MDS-UPDRS pt. III score	28.0	4-54	± 2.3	34.4	10-66	± 1.4
mH&Y stage	2.9	2.5-4	± 0.6	3.0	2.5-4	± 0.5
PEG-J duration (months)	7.7	0-27	± 5.9	5.7	0-27	± 4.9
**Reason for replacement**^c^	n	%		n	%	
Scheduled replacements	12	29.3		36	45.6	
Stoma complications^a^	6	14.6		9	11.4	
Unexpected replacements	29	70.7		43	54.4	
Stoma complications^a^	13	31.7		11	13.9	
Device related replacements	28			45		
Patient related replacements	3			8		
*Total*	31	75		53	67	

## Discussion

Gastrointestinal issues are frequent, up to 64% in PD patients [4]. Compared to a large review on DLI safety, [9] in a 12 months observation period, our results showed a similar rate of complications (respectively 47,2% and 46,7% of patients reported at least one device-related complication). The most common reported reasons for discontinuation are device-related adverse effects [10] and lack of effectiveness. [11] The former is the most common cause for DLI discontinuation (19.6%), [7, 10] suggesting that the adherence to DLI depends on PEG-J implant status. In our study, the rate of complications increased over time, and was associated to a higher number of replacement procedures. Device-related complications were common, although they were mild, not associated to worsening of motor scores, or discontinuation of infusion therapy (only one patient discontinued due to acute polyneuropathy). It is plausible that this result is related to the ease and rapidity of the “push” procedure. Moreover, a statistical comparison between “pull” and “push” technique did not show significant differences for age, disease duration, follow-up duration, PEG-J duration, UPDRS III and mH&Y scores.

The lower rate of stoma complications in the “push” group, associated to the lower rate of unexpected replacements, could be due to the higher turnover of replacements, that in turn may have acted as a protective factor against major complications. Any deterioration of the tubing system can reduce clinical benefit, increase the risk of stoma related complications or even cause sudden PEG-J breaking. Thus, a shorter PEG-J half-life, or higher turnover, together with an easy-to-use replacement technique may exert a protective role for device related complications. By reducing the number of operators needed to replace a device and simplifying endoscopic procedures, the “push” technique takes less time and is also less invasive. Finally, by not requiring sedation, it eliminates the risks of exposure to anesthetic drugs. The major limitation of this study was the retrospective nature and the low number of included patients.

## Conclusion

We found that the overall complication rate was lower for the “push” technique and this may have been due to its higher turnover rate, suggesting that it may have acted as a protective factor.

## Data Availability

All data analyzed during this study are included in this published article. If any additional data/files may be obtained from the corresponding author on reasonable request.

## Abbreviations

PEG-J: Percutaneous endoscopic gastrostomies with jejunal extension tubes
DLI: Duodenal levodopa infusion
APD: Advanced Parkinson’s disease
UPDRS: Unified Parkinson’s Disease Rating Scale
mH&Y: Modified Hoehn and Yahr scale
